# Patient-Centered Outcomes of an Emergency Department Social and Medical Resource Intervention

**DOI:** 10.5811/westjem.2022.10.57096

**Published:** 2022-12-21

**Authors:** Rohit Gupta, Anthony Wang, Daniel Wang, Daniela Ortiz, Karen Kurian, Thiago Halmer, Michael S. Jaung

**Affiliations:** * Baylor College of Medicine, School of Medicine, Houston, Texas; † Washington University in St. Louis, School of Medicine, St. Louis, Missouri; ‡ Duke University, School of Medicine, Durham, North Carolina; § Baylor College of Medicine, Department of Emergency Medicine, Houston, Texas

## Abstract

**Introduction:**

Few studies have examined the impact of emergency department (ED) social interventions on patient outcomes and revisits, especially in underserved populations. Our objective in this study was to characterize a volunteer initiative that provided community medical and social resources at ED discharge and its effect on ED revisit rates and adherence to follow-up appointments at a large, county hospital ED.

**Methods:**

We performed a cross-sectional analysis of ED patients who received medical and social resources and an educational intervention at discharge between September 2017–June 2018. Demographic information, the number of ED return visits, and outpatient follow-up appointment adherence within 30 and 90 days of ED discharge were obtained from electronic health records. We obtained data regarding patient utilization of resources via telephone follow-up communication. We used logistic regression analyses to evaluate associations between patient characteristics, reported resource utilization, and revisit outcomes.

**Results:**

Most patients (55.3% of 494 participants) identified as Latino/Hispanic, and 49.4% received healthcare assistance through a local governmental program. A majority of patients (83.6%) received at least one medical or social resource, with most requesting more than one. Patients provided with a medical or social resource were associated with a higher 90-day follow-up appointment adherence (odds ratio [OR] 2.56; 95% confidence interval [CI] 1.05–6.25, and OR 4.75; 95% CI 1.49–15.20], respectively), and the provision of both resources was associated with lower odds of ED revisit within 30 days (OR 0.50; 95% CI 0.27–0.95). Males and those enrolled in the healthcare assistance program had higher odds of ED revisits, while Hispanic/Latino and Spanish-speaking patients had lower odds of revisits.

**Conclusion:**

An ED discharge intervention providing medical and social resources may be associated with improved follow-up adherence and reduced ED revisit rates in underserved populations.

## INTRODUCTION

In the last two decades, the growth in the number of annual emergency department (ED) visits in the United States has outpaced the number expected by population growth by nearly two-fold.^1^,^2^ There has been a concomitant increase in the proportion of safety-net EDs serving high volumes of patients who are underinsured or enrolled in Medicaid.^3^,^4^ These trends are in part due to health inequities ingrained by social structures and economic systems, known as social determinants of health (SDoH).^5^ Both race/ethnicity and socioeconomic status have been strongly associated with disparities in attendance at safety-net hospitals as well as morbidity and mortality.^5^–^10^ Repeated ED utilization is also linked to higher mortality rates, especially in elderly patients.^11^ Patients with frequent ED revisits have limited connections to community resources and reduced comprehension of discharge instructions.^12^ Decreasing ED revisits may help alleviate high ED volumes, which are associated with increased in-hospital mortality, longer times to treatment initiation, and a higher likelihood of leaving against medical advice.^13^–^15^

There is a growing body of literature on the effectiveness of linking patients to primary care services from the ED and addressing SDoH to decrease hospital crowding.^16^,^17^ The ED is uniquely positioned to serve as a critical site to facilitate addressing social needs and promoting these linkages.^18^–^20^ For example, the Health Leads model and Highland Health Advocates both use help desks to connect patients to community-based resources from the ED; however, there remains a lack of evidence regarding how these approaches impact ED utilization outcomes.^21^, ^22^ Further, there is limited literature describing the utilization of social worker services, case management, and implementation of community interventions from an ED setting.^23^–^25^

Housing status, food insecurity, employment status, insurance status, education status, ability to pay for utilities, and availability of transportation are SDoH domains that can be targeted for intervention by multidisciplinary teams.^26^–^28^ While there are promising results from studies using vertical approaches that address one single SDoH domain, there are limited studies that have investigated the impact of programs that target multiple SDoHs.^29^,^30^ In this study we sought to assess a volunteer initiative that provided community medical and social resources at ED discharge and its effect on ED revisit rates and adherence to follow-up appointments at a large, county hospital ED.

## METHODS

### Study Design and Setting

We conducted a retrospective, cross-sectional study of ED patients at a large, county hospital (89,000 annual ED visits) in Houston, TX, who received a volunteer patient discharge intervention between September 1, 20171–June 1, 2018. This service was provided by a student-led organization of roughly 60 undergraduate volunteers from a nearby university. Texas did not expand Medicaid coverage under the Affordable Care Act, and most patients in this health system are underinsured or use a county financial assistance program (FAP) for medical services within the hospital system.^31^,^32^ This study received institutional review board approval.

### Intervention

Volunteers underwent biannual eight-hour trainings covering intervention procedures, resources provided to patients, and simulations of common patient encounters (Supplemental File 1). Spanish language competency of volunteers was assessed by native speakers. Teams of 3–4 volunteers with one supervising “shift leader” rotated from 1 pm-9 pm Monday to Saturday through a lower acuity treatment area for patients with an Emergency Severity Index of 3 or higher. The inclusion criterion was any patient marked for discharge in the care area displayed on the care area electronic board. Volunteers reviewed the patient with a nurse to confirm discharge status and to obtain the after-visit summary. Patients to be discharged to a skilled nursing facility, in-patient rehabilitation, or correctional facility were not approached. Low-acuity treatment areas were targeted as they had individual patient rooms with space for the volunteer teams to deliver the intervention and had a higher proportion of patients discharged compared to high-acuity areas.

**Population Health Research Capsule**

What do we already know about this issue?

*The ED is uniquely positioned to address patients’ social needs and promote linkages to community services, but limited evidence exists describing linkage models*.

What was the research question?

*Are health system utilization outcomes impacted if patients are provided community resources at ED discharge?*

What was the major finding of the study?

*Patients receiving resources had lower odds of ED revisit at 30 days and a higher 90-day follow-up appointment adherence*.

How does this improve population health?

*Providing resources upon ED discharge through a standardized process may reduce ED revisits and encourage outpatient follow-up*.

Patients who agreed to participate were asked questions from a standardized questionnaire to gather demographic information. Interventions were conducted in English or Spanish depending on patient preference. Patients were then provided a standardized educational intervention that involved reviewing their medication list and follow-up appointments and emphasizing the importance of medication and appointment adherence. Finally, patients were offered information on a variety of local and federal social and medical resources given in their preferred language. Resources were provided based on patients’ interest in receiving each resource. Medical resources included information on prescription discount cards, lists of pharmacies, primary care clinics, or low-cost dental clinics. Social resources included information on programs such as FAPs for rent, supplemental nutrition programs, and subsidized transportation programs. Each intervention lasted 5–15 minutes.

Patients were called one week after discharge by volunteers and asked questions from the standardized questionnaire regarding medication adherence, adherence at follow-up appointments, and utilization of resources that they received in the ED. Two additional attempts were made to reach patients who did not answer the first call at 30 minutes and again at one week after.

### Data Collection

Patient responses during the intervention and follow-up calls were recorded using standardized forms. Additional patient information including demographics, ED chief complaint, and outcome variables was obtained from electronic health records (EHR) and recorded in a standardized tool. We used the patients’ listed ZIP codes as a proxy for socioeconomic status,^33^ and median household income data was obtained from the 2013–2017 American Community Survey.^34^ Data was de-identified and stored in a secure database.

### Outcomes

The primary outcome was the frequency of ED revisits to any Harris County-funded hospital, with a secondary outcome of adherence to follow-up clinic appointments. Revisits and appointment adherence were evaluated within 30 and 90 days after initial ED discharge, as prior studies have used these times as endpoints, and more than 30 days may be required to enroll or experience impact from new services.^35^–^37^ The 90-day outcomes were inclusive of ED revisits and appointment attendance within the initial 30 days.

### Analysis

Patients who were less than 18 years of age or pregnant at the time of the intervention were excluded from data analysis. We also excluded patients with missing identifying information on the standardized forms. Patient characteristics and outcomes were analyzed using descriptive and inferential statistics. We used binomial logistic regression to assess the relationship among independent variables (patient demographics, type of resources provided at ED discharge, and reported resource utilization at follow-up call) and dependent variables (follow-up appointment adherence and ED revisits), using SPSS Statistics for Windows, version 26 (IBM Corp., Armonk, NY). We performed a residuals analysis to identify outliers with standardized residuals greater than 2.5 standard deviations, which were removed from the final analysis.

## RESULTS

### Characteristics of Study Subjects

A total of 614 patients received the intervention during the study period (Figure). Patients below 18 years of age (104), pregnant at the time of discharge (7), or with missing medical record numbers or ED visit dates (9) were excluded. We included a final 494 patient encounters in the data analysis. The median patient age was 43 years (Table 1). Most patients were female (55.3%), and the majority identified as Latino/Hispanic (55.3%). Primary Spanish speakers made up over one third (35.2%) of all patients. The most frequent chief complaints were abdominal pain (19.6%), generalized pain (8.5%), and headache (6.1%). About half of the patients (49.4%) were enrolled in the county healthcare FAP. We found that 33.4% of patients were uninsured, and only 13.6% had insurance coverage. These characteristics overall reflected the general ED population at this hospital.^31^

### Main Results

A total of 413 patients (83.6%) requested at least one resource at discharge, with 329 (66.6) requesting more than one resource. The most requested medical and social resources were dental care information and information on food and insurance assistance, respectively (Table 2). From 494 ED encounters included in this study, volunteers contacted 158 patients (32%)in a follow-up call one week after discharge. Compared to patients who were not successfully contacted, this patient population did not significantly differ in gender (*P* = 0.29), race/ethnicity (*P* = 0.18), language (*P* = 0.89), or insurance status (*P* = 0.12). Of the contacted patients, 81 (51.3%) reported using a resource received from the intervention. Of all patients, 76 (15.4%) returned to the ED at least once within 30 days of discharge, and 114 (23.1%) returned within 90 days.

Components of our intervention were associated with improved outcomes of decreased odds of ED revisits and improved attendance of follow-up appointments (Table 3). Patients who requested both medical and social resources from the intervention was associated with lower odds (odds ratio [OR] 0.50, 95% confidence interval [CI] 0.27–0.95) of an ED revisit at 30 days compared to those requested no resources. Those who reported using a resource received from the intervention (OR 0.46, 95% CI 0.24–0.92) had lower odds of revisiting at 90 days. There were higher odds of outpatient follow-up appointment adherence for patients who received a social resource at discharge (OR 4.75, 95% CI 1.49–15.20), and those who received a medical resource (OR 2.56, 95% CI 1.05–6.25).

We observed a difference in the odds of ED revisits and attendance of follow-up appointments associated with some patient characteristics. Increased odds of an ED revisit within 30 days of discharge were seen in males (OR 1.76, 95% CI 1.07–2.88) and patients enrolled in the county FAP (OR 2.11, 95% CI 1.15–3.87). Males also had higher odds (OR 1.91, 95% CI 1.25–2.91) of revisiting at 90 days. Patients in the 3^rd^–5^th^ quintile median household income had lower odds of attendance to follow-up appointments within 30 days of ED discharge (OR 0.38, 95% CI 0.16–0.90).

In contrast, primarily Spanish speakers had lower odds of an ED revisit (OR 0.53, 95% CI 0.33–0.85) and higher odds of attending at least one follow-up appointment at 30 and 90 days. Hispanic/Latino patients had lower odds of revisiting the ED within 90 days compared to Black patients (OR 0.52, 95% CI 0.33–0.83) as well as higher odds of follow-up attendance at 30 and 90 days. Patients enrolled in a county FAP also had higher odds of follow-up attendance compared to uninsured patients.

## DISCUSSION

Our findings indicate that ED discharge interventions focused on patient needs and providing social and medical resources may assist in promoting appropriate patient access to the healthcare system after ED discharge. The most requested resources were information on local dental, primary care, and pharmacy services, as well as food and health insurance resources. Similar needs were identified in surveys of ED patients who made early or frequent returns to the ED after their initial ED discharge.^38^,^39^ These patients reported difficulty scheduling a primary care appointment, attending outpatient appointments due to lack of insurance, and finding transportation to attend follow-up appointments.^38^,^39^

In our study, patients who requested both social and medical resources had lower rates of adherence to follow-up compared to those who requested only one category of resources, possibly indicating that patients with multiple needs had more barriers to appointment adherence. Furthermore, patients reported the discharge process of their initial ED visit was rushed, unprepared, and left them confused.^38^ Our volunteer-led service was designed to address these factors more comprehensively during ED discharge.

Despite identified patient needs, interventions dedicated to providing SDoH resources are sparse. Wassmer et al described using a peer counseling program that provided education on medical and social needs in the ED.^40^ Patients who had visited the ED four or more times in the previous year were counseled during their ED visit and in subsequent visits, with a decrease in ED utilization over two years extending past the follow-up period of the study.

A population-based approach to ED social interventions may improve the effectiveness of addressing SDoH by identifying risk factors for ED revisits and developing interventions to target specific population needs. This study found that male gender, Black race, and use of the county FAP were associated with increased odds of in-system ED revisits. Other studies have reported mixed results on the association between these factors and ED usage. One study found an association between male gender and higher ED revisit rates in older adults.^11^ However, others demonstrated no such association or an inverse association,^41^–^44^ which likely demonstrates that the impact of gender may be influenced by other risk factors. Multiple studies have demonstrated higher ED revisit rates among Blacks compared to other ethnic groups; however, this may be due to differences in average income, enrollment in Medicare and Medicaid, implicit bias against this group within medical systems, and lack of access to primary care physicians.^39^,^44^,^45^

The impact of using a healthcare FAP for addressing healthcare costs has not been well characterized. Similar to the findings in this study, Wassmer et al found that patients receiving financial assistance from a county program in California had higher utilization of the ED,^40^ which was speculated to be due to younger, lower income patients on financial assistance than those enrolled in public insurance programs. Interestingly, although the use of a county FAP was associated with increased odds of ED revisit, this was also associated with increased odds of follow-up appointment attendance at 90 days post-discharge. Possibly, the cost of appointments is ameliorated by the assistance program, and for similar reasons these patients receiving financial assistance may be less deterred from revisiting the ED.

Our study differed from preceding literature on the impact of English proficiency. Ngai et al demonstrated that patients with limited English proficiency have a higher likelihood of an unplanned ED visit within 72 hours of ED discharge compared to English speakers, even after adjusting for potential confounders.^46^ The opposite trend was observed in this study, with lower odds of a return to the ED within 90 days in primary Spanish speakers. The reason for this is likely multifactorial. Previous studies suggest that less acculturated Hispanic adults, measured by citizenship status and length of stay in the US, use fewer healthcare resources overall than more acculturated counterparts, and those who are undocumented may fear discovery and deportation, avoiding ED use for non-urgent reasons.^47^,^48^ Finally, having a higher median income was significant for lower odds of follow-up appointment adherence, but not a significant risk factor for ED revisits. Previously, lower socioeconomic status has been established as a risk factor for increased ED utilization, but its impact on appointment adherence has been debated.^3^,^49^

Dedicated personnel in the ED setting are likely needed to effectively attend to patients’ overlapping medical and social gaps. Many healthcare organizations employ ED social workers, case managers, and patient navigators who address the impact of SDoH through patient counseling, referrals to community services, and patient discharge planning.^50^ The advantage provided by this personnel is supported by multiple systematic reviews demonstrating that their work reduces ED revisits.^24^,^51^ However, a social worker-based intervention may not be feasible at all hospitals, which may be understaffed in high-volume, safety-net facilities treating patients with complex medical and social problems.^27^

Our study explored the possibility of using trained volunteers to perform an educational intervention. The Health Leads models similarly used volunteer patient advocates to connect patients with social resources.^21^ Recruiting volunteers for our intervention allowed for more patients to be educated on available resources. Such a model may be scalable to other hospital settings, as implementation required minimal training of volunteers and an upfront investment of time to collect information about county and federal resources. In our experience, this investment was associated with a reduction of ED revisits similar to that seen in complex care coordination systems, suggesting that dedicated volunteers may serve as an adequate patient navigator proxy. Further studies are warranted to examine the impact volunteers and such ancillary staff has on patient outcomes.

## LIMITATIONS

As this study used a retrospectively reviewed cross-section of patients’ phone interviews and EHRs, causation cannot be inferred between the intervention and revisits or follow-up adherence. This was a single-site study at a county ED assessing patients at low-acuity units; therefore, our findings may not be generalizable to other ED settings. We were unable to collect data on a control cohort of patients who did not receive this intervention due to resource-limitations, and we did not calculate the proportion of participants of all ED patients triaged to these acuity areas during the study period. Most patients in this study were either uninsured or used a county FAP covering care for in-system healthcare services only, and there was no method to track out-of-system healthcare encounters after discharge.

We used convenience sampling to select patients during times when volunteers were present in the ED. Patients discharged during late evening or morning hours were not included, which may have skewed the characteristics of the population studied. ZIP code data was used as a proxy for socioeconomic status and may not have been representative of each patient’s income. Recall bias may be introduced via patient self-reporting of usage of medical and social resources during the follow-up call. Non-response bias may have been introduced as only one follow-up call was made, and further follow-up calls were constrained by available resources, but we did not observe a significant difference between patients who were and were not reached.

## CONCLUSION

The outcomes from this intervention suggest that there is an opportunity to improve patient engagement with the healthcare system by providing resources that address social determinants of health. This suggests that a standardized in-person approach may reduce ED revisits and improve outpatient follow-up. Future investigation is needed to examine the best methods for implementation, comparing in-person and non-individualized interventions, and cost effectiveness of programs to address SDoH in the ED that meet patients’ social needs and promote healthcare accessibility.

## Supplementary Information



## Figures and Tables

**Figure:**
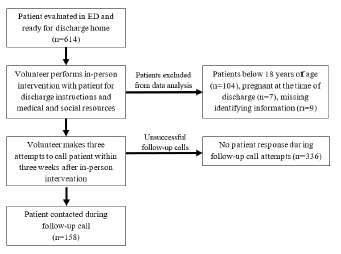
Educational intervention workflow showing the steps performed when discharging and following up with patients. *ED*, emergency department.

**Table 1:** Characteristics of patients who received intervention.

Characteristic	Number (%) / median (IQR)
Age (median years)	43 (31 – 53)
Gender	
Female	273 (55.3)
Male	221 (44.7)
Race/ethnicity	
Black	152 (30.8)
White	48 (9.7)
Hispanic/Latino	273 (55.3)
Other	21 (4.3)
Preferred language	
English	316 (64.0)
Spanish	174 (35.2)
Other	2 (0.4)
Unknown	2 (0.4)
ZIP code household median income quintile	
1st quintile ($26,644 – $47,297)	290 (58.7)
2nd quintile ($47,297 – $69,446)	146 (29.6)
3rd–5th quintiles ($69,446 – $180,758)	53 (10.7)
Unknown	5 (1.0)
Insurance status	
Uninsured	165 (33.4)
County financial assistance program	244 (49.4)
Public/private insurance	67 (13.6)
Unknown	18 (3.6)
Resource requested	
No resources	81 (16.4)
Social resources only	71 (14.4)
Medical resources only	88 (17.8)
Both resources	254 (51.4)
Resources used as reported on follow-up call	
Not reached by phone	336 (68.0)
Reached by phone and did not use resources (or no resources given)	77 (15.6)
Reached by phone and reported resource use	81 (16.4)
Outcomes	
Any ED revisit within 30 days	76 (15.4)
Number of ED revisits within 30 days (median visits)	1 (1)
Any ED revisit within 90 days	114 (23.1)
Number of ED revisits within 90 days (median visits)	1 (1 – 2)
Attendance of follow-up appointment within 30 days	185 (72.5)
Attendance of follow-up appointment within 90 days	240 (75.0)

*IQR*, interquartile range; *ED*, emergency medicine.

**Table 2:** Most common medical and social resources requested by patients through the intervention.

Resource	Number given (% of total patients)
Top 5 medical resources given	
Low-cost dental clinic information	216 (43.7)
Primary care clinic information	205 (42.0)
List of local pharmacies	147 (29.8)
Information card for local medical insurance	126 (25.5)
Prescription discount card	122 (24.6)
Top 5 social resources given	
General information sheet on food and insurance assistance	234 (47.4)
Information on local financial and utility bill assistance	61 (12.3)
List of homeless shelters and emergency housing options	59 (11.9)
Information on English as a second language courses	58 (11.7)
Application for local transportation assistance services	49 (9.9)

**Table 3:** Logistic regression analysis of 30- and 90-day follow-up appointment attendance and emergency department revisit.

Characteristic	30-day ED revisit OR (95% CI)	90-day ED revisit OR (95% CI)	30-day follow-up appointment attendance OR (95% CI)	90-day follow-up appointment attendance OR (95% CI)
Gender				
Female	Reference			
Male	*1.76 (1.07–2.88)	*1.91 (1.25–2.91)	0.83 (0.48–1.44)	0.83 (0.50–1.38)
Race/ethnicity				
Black	Reference			
Hispanic/Latino	0.62 (0.36–1.07)	*0.52 (0.33–0.83)	*2.86 (1.52–5.40)	*3.29 (1.86–5.83)
White	0.72 (0.30–1.78)	0.98 (0.48–2.00)	0.62 (0.25–1.57)	2.10 (0.81–5.41)
Preferred language				
English	Reference			
Spanish	0.72 (0.42–1.23)	*0.53 (0.33–0.85)	*2.00 (1.12–3.57)	*2.56 (1.4–4.50)
ZIP code median household income quintile				
1st Quintile	Reference			
2nd Quintile	0.97 (0.55–1.70)	0.93 (0.58–1.51)	1.03 (0.55–1.92)	0.73 (0.42–1.29)
3rd–5th Quintiles	1.50 (0.7–3.15)	1.64 (0.86–3.10)	*0.38 (0.1–0.90)	0.47 (0.2–1.03)
Insurance status				
Uninsured	Reference			
Public/private Insurance	1.26 (0.51–3.11)	1.41 (0.70–2.85)	0.68 (0.28–1.65)	0.57 (0.25–1.28)
County financial assistance program	*2.11 (1.15–3.87)	1.63 (0.99–2.69)	*2.01(1.03–3.91)	*1.89 (1.02–3.50)
Resources requested				
No resources	Reference			
Social resources	0.60 (0.26–1.36)	0.65 (0.31–1.35)	3.28 (1.15–9.36)	*4.75 (1.49–15.20)
Medical resources	0.52 (0.23, 1.14)	0.54 (0.2–1.09)	2.48 (0.97–6.31)	*2.56 (1.0–6.25)
Both	*0.50 (0.27–0.95)	0.63 (0.3–1.11)	1.63 (0.8–3.26)	1.23 (0.65–2.33)
Resources used as reported on follow-up call				
Not reached by phone	Reference			
Reached by phone and did not use resource	0.83 (0.41–1.68)	0.90 (0.5–1.61)	1.42 (0.66–3.09)	1.43 (0.67–3.04)
Reached by phone and reported resource use	0.63 (0.30–1.32)	*0.46 (0.24–0.92)	1.00 (0.46–2.16)	0.94 (0.48–1.87)

* P < 0.05.

*CI*, confidence interval; *OR*, odds ratio.
