# Supplementary material for: Patient-Centered Outcomes of an Emergency Department Social and Medical Resource Intervention
Source: West J Emerg Med. 2022 Dec 21;24(2):193–200. doi: 10.5811/westjem.2022.10.57096 (PMC10047735; doi:10.5811/westjem.2022.10.57096)

**Supplemental File 1**

**Patient Discharge Initiative Training and Programmatic Materials**

**Biannual Training Summary**

- Format: In-person lectures and small-group sessions over course of one day
- Welcome, Patient Discharge Initiative (PDI) mission statement, and history of PDI
- Background on intervention hospital and emergency department
- Hospital volunteer registration requirements, dress code, what to bring, and directions
- Hospital rules including protection of confidentiality and privacy, how to respond when patients request medical advice
- Structure of ED, activity areas, and shift schedules
- General volunteer shift information
- Protocol details
  - Approaching patient
  - Patient enrollment
  - Documentation of intervention
  - Full resource list review
  - Discharge at the end of the intervention
  - Follow up calls
- Special considerations of working in the ED waiting room
- Review of typical patient intervention
- Small group sessions covering topics
  - New resources walkthrough
  - Discharging the Spanish-speaking patient
  - Waiting room procedures and follow up calls
  - Insurance and medications
  - Medical and social resources
- Practice small group scenarios reviewing possible difficult patient interactions

**Estimated Costs:**

| Transportation cards for participants (150) | $375 |
| --- | --- |
| Food for biannual training | $200 |
| Miscellany office supplies | $50 |
| Total | $625 |

**Intervention Documentation Note:**


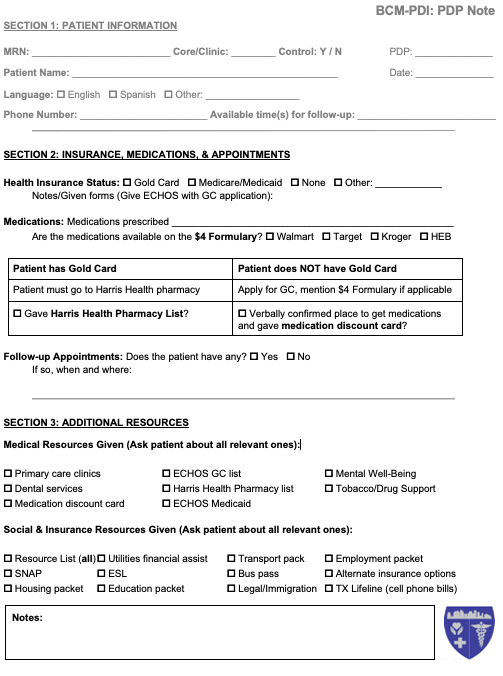

Supplement: Supplementary file 1 [file wjem-24-193-s001.docx]
